# Imprecision of Adaptation in *Escherichia coli* Chemotaxis

**DOI:** 10.1371/journal.pone.0084904

**Published:** 2014-01-08

**Authors:** Silke Neumann, Nikita Vladimirov, Anna K. Krembel, Ned S. Wingreen, Victor Sourjik

**Affiliations:** 1 Zentrum für Molekulare Biologie der Universität Heidelberg (ZMBH), DKFZ-ZMBH Alliance, University of Heidelberg, Heidelberg, Baden-Württemberg, Germany; 2 Department of Molecular Biology, Princeton University, Princeton, New Jersey, United States of America; Centre National de la Recherche Scientifique, Aix-Marseille Université, France

## Abstract

Adaptability is an essential property of many sensory systems, enabling maintenance of a sensitive response over a range of background stimulus levels. In bacterial chemotaxis, adaptation to the preset level of pathway activity is achieved through an integral feedback mechanism based on activity-dependent methylation of chemoreceptors. It has been argued that this architecture ensures precise and robust adaptation regardless of the ambient ligand concentration, making perfect adaptation a celebrated property of the chemotaxis system. However, possible deviations from such ideal adaptive behavior and its consequences for chemotaxis have not been explored in detail. Here we show that the chemotaxis pathway in *Escherichia coli* shows increasingly imprecise adaptation to higher concentrations of attractants, with a clear correlation between the time of adaptation to a step-like stimulus and the extent of imprecision. Our analysis suggests that this imprecision results from a gradual saturation of receptor methylation sites at high levels of stimulation, which prevents full recovery of the pathway activity by violating the conditions required for precise adaptation. We further use computer simulations to show that limited imprecision of adaptation has little effect on the rate of chemotactic drift of a bacterial population in gradients, but hinders precise accumulation at the peak of the gradient. Finally, we show that for two major chemoeffectors, serine and cysteine, failure of adaptation at concentrations above 1 mM might prevent bacteria from accumulating at toxic concentrations of these amino acids.

## Introduction

Adaptation is an important property of many sensory systems that allows them to recover from an initial stimulation and to regain activity and responsiveness even at high levels of persistent stimulation. One of the most analyzed models for adaptation in cell signaling is bacterial chemotaxis, where the recovery from the initial attractant or repellent stimulation is mediated by changes in the methylation levels of chemoreceptors [1]–[5]. Receptors are methylated or demethylated on four to five specific glutamate residues by the methyltransferase CheR and the methylesterase CheB. CheR preferentially recognizes the inactive state of the receptors and increases receptor activity through methylation, thus countering the effects of chemoattractants that inhibit receptor activity. CheB preferentially demethylates active receptors and thereby lowers their activity upon removal of attractants or addition of repellents. In the absence of a gradient, these feedbacks ensure that receptor methylation and therefore activity of the receptor-associated kinase are adjusted to generate intermediate levels of the phosphorylated response regulator CheY (CheY-P). CheY-P binds to flagellar motors and induces a switch in the direction of motor rotation that results in cell tumbling and reorientation. As a consequence, an intermediate level of CheY-P that falls into the narrow working range of the motor [6] results in an intermediate switching rate and produces a random sequence of runs and tumbles. This allows cells to explore their environment and, importantly, by the suppression of tumbles, to respond sensitively to an increase in attractant concentration thus yielding longer runs in that favorable direction [7], [8].

High precision of adaptation in the presence of ambient ligand is commonly assumed to be an essential feature of chemotaxis [9]–[11], because it ensures that the level of CheY-P, and as a consequence the steady state tumbling bias of the cell, are adjusted within an optimal range for chemotaxis [12]. However, already an early study of *Escherichia coli* chemotaxis [8] had reported highly imprecise adaptation to high concentrations of serine, a ligand sensed by the major receptor Tsr. Imprecise adaptation to several other attractants, including high concentrations of aspartate or its non-metabolizable analogue α-methyl-DL-aspartate (MeAsp) that are sensed by another major *E. coli* receptor Tar, has also been confirmed by more recent studies [13]–[15].

In this work, we aimed to better understand both the limits of precise adaptation in *E. coli* chemotaxis and the importance of imprecise adaptation. We show that adaptation becomes increasingly imprecise at high ligand concentrations, most likely due to saturation of available methylation sites. Using computer simulations we show that a limited precision of adaptation has little effect on the rate of the chemotactic movement in a gradient, but reduces the ability of bacteria to accumulate at exactly the peak of the gradient. Moreover, we speculate that the large imprecision of adaptation for some amino acids may benefit bacteria, by preventing their accumulation at toxic concentrations that are inhibitory to cell growth.

## Results and Discussion

### Precision of Adaptation Towards Different Chemoattractants

**Figure 1:**
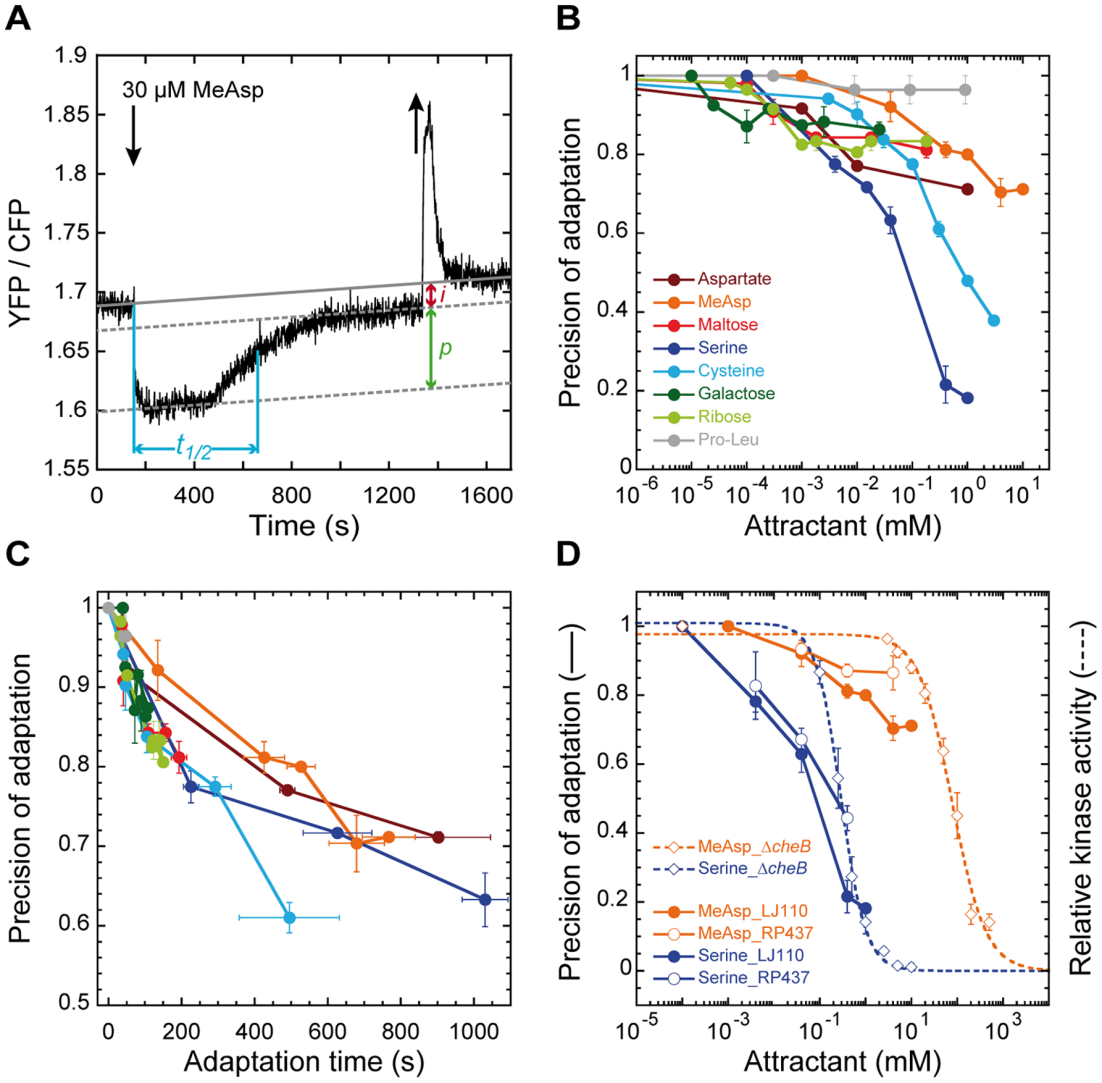
Precision of adaptation in wild-type cells. (**A**) Example FRET measurement of the pathway response. Cells expressing the CheY-YFP/CheZ-CFP FRET pair, a reporter for kinase activity, were stimulated by step-like addition (down arrow) and subsequent removal (up arrow) of 30 µM MeAsp, a nearly saturating stimulus. Changes in kinase activity in response to addition of attractant were used to determine adaptation time (*t_1/2_*), defined as the time required to regain 50% of the initial loss in activity, and imprecision of adaptation (*i*), defined as the difference in the YFP/CFP ratio between cells adapted to buffer (interpolated grey solid line) and to a given attractant concentration (parallel dotted line below). Precision of adaptation (*p*), the fraction of kinase activity recovered in the presence of ambient ligand, was then obtained as 1−*i*, with 1 being the maximum possible precision (see *Materials and Methods* for details). The upward drift of the baseline is due to faster photobleaching of CFP compared to YFP. (**B**) Dependence of precision of adaptation on attractant concentration in wild-type strain SN1 [LJ110 Δ(*cheY cheZ*)]. Values were normalized to the response of buffer-adapted cells to a saturating ligand concentration. Some data in (B) were replotted from [14]. Error bars here and throughout indicate standard errors for multiple measurements. (**C**) Dependence of precision of adaptation on adaptation time. (**D**) Comparison of precision of adaptation for MeAsp (orange symbols) and serine (blue symbols) in strains SN1 (closed circles) and VS104 [RP437 Δ(*cheY cheZ*), open circles] with dose-response curves of *cheB* strain VS124 [RP437 Δ(*cheB cheY cheZ*)] for steps of MeAsp or serine (open diamonds). Relative kinase activity was calculated as described previously [16], [17] and fitted by a multisite Hill equation [17].

We first systematically analyzed the precision of adaptation over a range of concentrations for several attractants that are sensed by Tsr (serine, cysteine), Tar (aspartate, MeAsp, maltose) or by the minor receptors Trg (galactose, ribose) and Tap (proline-leucine dipeptide; Pro-Leu). We used a FRET-based assay that relies on the phosphorylation-dependent interaction of CheY fused to yellow fluorescent protein (CheY-YFP) with its phosphatase CheZ fused to cyan fluorescent protein (CheZ-CFP). It provides a direct readout of the pathway kinase activity and allows us to follow the initial response as well as the subsequent adaptation [16], [17], from which adaptation time and precision can be determined (Fig. 1A). For all tested ligands a gradual decrease in the precision of adaptation, indicated by a lower adapted activity compared to the prestimulus kinase activity, was observed with increasing strength of the initial stimulus (Fig. 1B). This decrease had different concentration dependence for individual ligands likely due to differences in ligand affinities. However, when the precision of adaptation was plotted as a function of the adaptation time *t_1/2_*, i.e. the time required to regain 50% of the initial pathway activity (Fig. 1A), precision values for most ligands aligned closely, especially for stimuli eliciting adaptation times shorter than 200 s (Fig. 1C). Adaptation time is known to be directly proportional to the stimulus strength, i.e. to the change in receptor occupancy or more exactly in the free-energy of receptor complexes elicited by a particular concentration of ligand [14], [18], because more receptor methylation is required to offset a stronger stimulus. Similar correlation between adaptation imprecision and time for most ligands therefore suggests a relatively uniform loss in precision with stimulus strength. Note that smaller imprecision and shorter adaptation time for sugars and dipeptides at saturating concentrations can be explained by the generally lower signaling strength of these ligands [14]. Interestingly, precision of adaptation for the two directly binding ligands of Tar, aspartate and MeAsp, was somewhat higher than for other ligands. This is consistent with the previously reported high precision of adaptation to aspartate [8], [10], [15] but suggests that such high precision represents the exception rather than the rule.

### Causes of Imprecise Adaptation

The observed gradual loss of precision might be due to the inability of receptor methylation to compensate for stimulation-dependent changes in receptor activity as the receptors approach saturation of available modification sites. To test this, we determined the response of a *cheB* strain [16] to addition of attractant (MeAsp and serine; Fig. 1D). This strain is characterized by a high modification state of receptors because CheR-mediated receptor methylation is not counterbalanced by CheB. In addition to its methylesterase activity, CheB also functions as a deamidase, irreversibly converting nonmethylatable glutamine (Q) residues into glutamates (E). Wild-type receptors are synthesized with a QEQE pattern at the four major methylation sites [19]. Since glutamines are functionally similar to methylated glutamates (E_m_), the predominant receptor modification state in the *cheB* strain, QE_m_QE_m_, approximates the upper limit on the possible recovery of kinase activity at a given ambient ligand concentration [20]. In the high concentration range, the failure of precise adaptation for both MeAsp and serine indeed correlated well with the dose-response curves for *cheB* cells (Fig. 1D), but imprecise adaptation was already observed at attractant concentrations lower than those inhibiting fully modified receptors. Therefore, approaching the level of saturation of methylation sites impairs precise adaptation. This observation is consistent with previous theoretical studies [13], [21], which suggested that reduction of the rate of receptor methylation due to the limiting number of available methylation sites leads to violation of the conditions for precise adaptation. Similar dependencies were observed for the two different *E. coli* strains LJ110 and RP437 (Fig. 1D), despite differences in the levels and ratios of receptor expression between these strains [14].

### Consequences of Imprecise Adaptation for Navigation in Chemical Gradients

**Figure 2:**
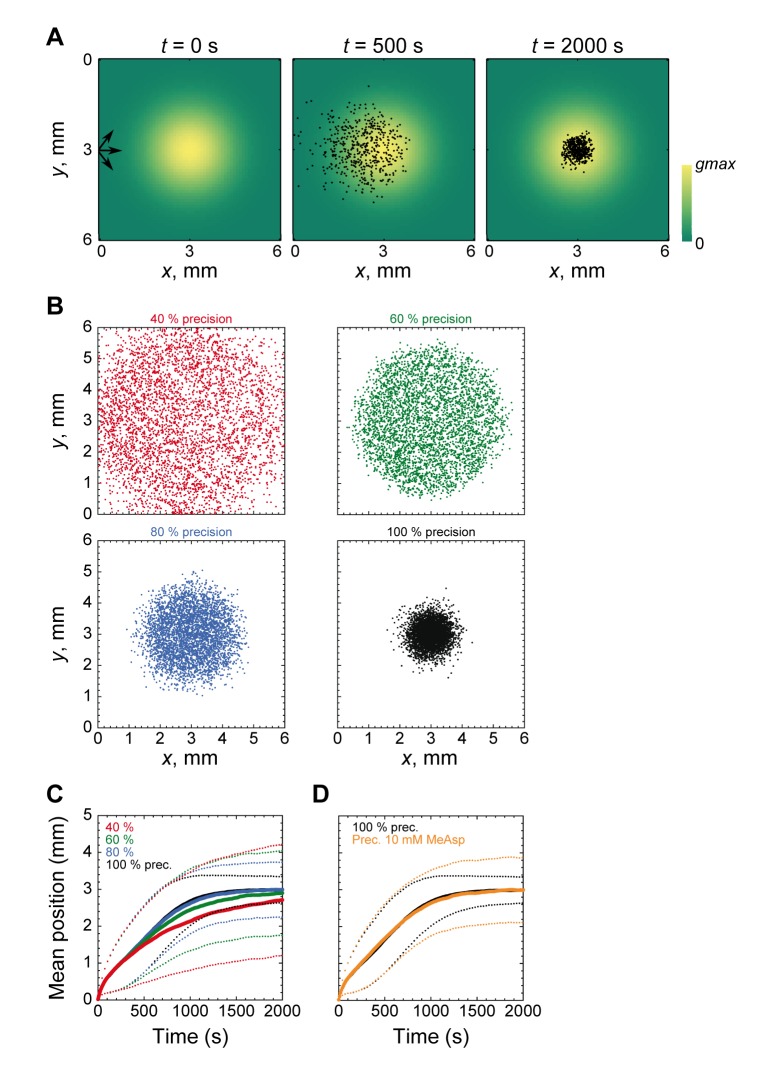
Simulated effects of imprecise adaptation on chemotactic behavior. (**A**) Cartoon illustrating the simulation of chemotaxis in a radially symmetric Gaussian gradient of attractant. Color scale indicates attractant (MeAsp) concentration from zero to the peak of the gradient (*gmax* = 10 mM MeAsp). Cells (dots) were initially placed at the left side of the profile, and the mean *x*-position of the population <*x*(*t*)> as well as the distribution of *x*-positions of all individual cells around the mean were followed for 2000 s. (**B**) Positions of populations of cells with precise adaptation or with various levels of decreased precision. (**C**) Mean position <*x*(*t*)> as function of time for cells with precise or fixed precision as in (B) and (**D**) for cells with a concentration-dependent precision of adaptation adapted from Fig. 1B. Simulation was performed in a gradient with *gmax = *10 mM MeAsp. Simulations at other peak concentrations are shown in Fig. S1. Solid lines in (C, D) indicate the mean <*x*(*t*)>, thin dotted lines indicate the distribution of the population around the mean, <*x*(*t*)>− s. d. and <*x*(*t*)>+ s. d., respectively.

Motivated by our experimental observations, we performed computer simulations to investigate possible consequences of imprecise adaptation on the chemotactic behavior of *E. coli* cells in chemical gradients. Therefore, we simulated chemotactic cell movement using RapidCell [12] in a Gaussian-shaped profile of attractant (MeAsp) with a peak concentration of 10 mM (Fig. 2A). We first investigated the effects of lowering the precision of adaptation to a fixed value independent of the ambient attractant concentration (Fig. 2B, C). Surprisingly, we observed that decreasing the precision of adaptation up to 60% had little effect on the chemotactic drift velocity up a gradient (Fig. 2C, solid lines). High drift velocities apparently persist because cells are still chemotactic and capable of following gradients as long as the CheY-P concentration remains in the working range of the flagellar motor [6], allowing the adapted cells to both run and tumble and therefore to respond to positive and negative stimulation.

Interestingly, imprecise adaptation had a much stronger effect on the ability of cells to accumulate exactly at the peak of the attractant profile, another parameter characterizing the chemotactic behavior of the cell population. Here, an even modest decrease in the precision of adaptation substantially broadened the distribution of cell positions around the peak of attractant (Fig. 2B and Fig. 2C, dashed lines). The same results were obtained when the precision of adaptation in the simulation was reduced as a function of attractant concentration, as observed experimentally for MeAsp (Fig. 2D), with cells traveling up the gradient of attractant at a nearly identical rate as precisely adapting cells (Fig. 2D, solid lines) but showing a broader distribution around the profile at equilibrium (Fig. 2D, dashed lines). A similar effect was observed at 0.1, 1 and 100 mM MeAsp peak concentration, although the difference became smaller at lower peak concentrations where the precision of adaptation is less affected (Fig. S1). Taken together, we conclude that moderately imprecise adaptation might be well tolerated by cells performing chemotaxis in transiently existing gradients, where precise accumulation at the peak is not important. Such transient gradients may exist, for example, in the mammalian gut or in the aquatic environment [22].

### Limits of Adaptability and Attractant Metabolism

**Figure 3:**
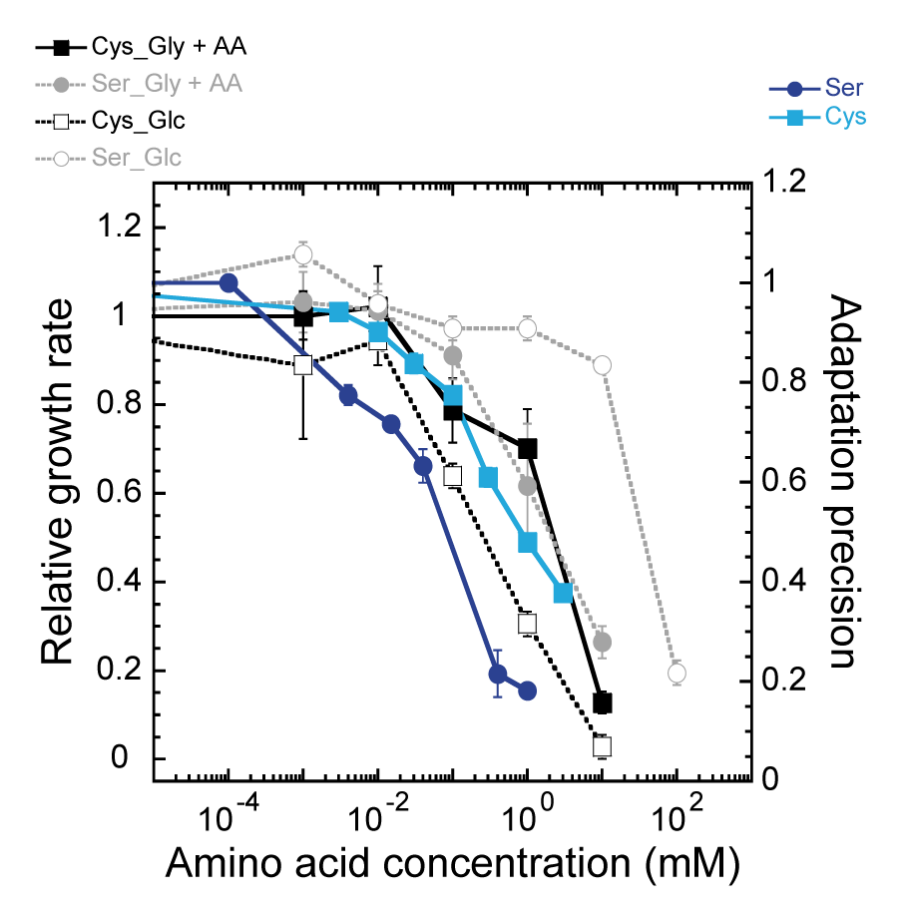
Inhibition of *E. coli* growth by serine and cysteine. *E. coli* MG1655 cells were grown at 34°C in M9 minimal medium [29] supplemented with 10 µg/ml thiamine and either 0.4% glycerol and four amino acids (Gly+AA) or 0.4% glucose (Glc). Indicated amounts of L-serine or L-cysteine were added after 2 hours of growth. Relative growth rate was determined as the difference between the optical density of the culture at 600 nm after 3.5 h and 5 h of growth, normalized to the value of a control culture grown without serine or cysteine. Adaptation precision for serine and cysteine was replotted from Fig. 1B.

Despite the apparent ability of chemotactic cells to tolerate modestly imprecise adaptation, it is obvious that precision of less than about 60% severely compromises the accumulation of bacteria at a peak of attractant, by making cells almost exclusively smooth swimming and therefore poorly able to sense gradients (Fig. 2B, C). Experimentally, such high imprecision is particularly apparent for the chemotactic response to serine and cysteine (Fig. 1B), the two primary ligands of Tsr [23], limiting the ability of *E. coli* to efficiently follow gradients of these amino acids at concentrations above about 0.1 mM. Interestingly, such limited range for chemotaxis appears to correlate with the previously reported negative effects of high concentrations of these two amino acids on cell metabolism and growth [24]–[27]. We confirmed that concentrations of serine and cysteine above 0.1 mM reduced *E. coli* growth in minimal medium (Fig. 3), whereas other attractant amino acids had no effect (Fig. S2 A). Despite some strain- and medium-specific differences (Figs. 3 and S2 B), the growth inhibition occurs in the same concentration range where the precision of adaptation strongly deteriorates. It is thus possible that the imprecise adaptation mediated by Tsr was evolutionarily selected to prevent bacterial accumulation at growth-inhibitory concentrations of these ligands to avoid their interference with synthesis of other amino acids.

## Materials and Methods

### Culture Conditions

For all experiments, *E. coli* K-12 strains were grown at 34°C and 275 rpm. For adaptation measurements cells were prepared as described in detail in [14]. Briefly, cultures were diluted 1∶100 from overnight cultures and grown to OD_600_ of 0.45 in Tryptone Broth supplemented with 50 µM IPTG to induce expression of the CheY-YFP/CheZ-CFP FRET pair from pVS88 [28]. Harvested cells were resuspended in tethering buffer (10 mM KPO_4_, 0.1 mM EDTA, 1 µM methionine, 10 mM lactic acid, 67 mM NaCl, pH7) and kept at 4°C.

For growth assay, cells were grown in M9 minimal medium [29] supplemented with 10 µg/ml thiamine and either 0.4% glycerol and four amino acids (40 µg/ml L-threonine, L-methionine, L-histidine, L-leucine) or 0.4% glucose.

### Adaptation Measurements and Data Analysis

Cells were attached to a polylysine-coated coverslip and kept under a constant flow of tethering buffer at a rate of 300 µl/min in a flow chamber. To add or remove attractant, the attached syringe pump was stopped briefly. Duration of adaptation to a single stimulus was up to 30–45 min, significantly longer than the measured adaptation times. After this time, no significant changes in adaptation could be observed at high ligand concentrations. Adaptation time, *t_1/2_*, was determined at the point of half-maximal recovery of the initial loss in kinase activity in response to attractant stimulation (see Fig. 1A). Adaptation imprecision (*i*), defined as the difference between the YFP/CFP ratio in the buffer and in the presence of ambient ligand, was calculated and normalized to the amplitude of response to a saturating stimulus of attractant (100 µM MeAsp), to allow for comparison of independent measurements. Precision of adaptation was then derived from these data as *p* = 1−*i*.

Excitation of CFP, the FRET donor, results in faster photobleaching in the cyan channel [13], [14], and as a consequence the YFP/CFP ratio during the measurement constantly increases in a nearly linear fashion [30]. We accounted for this drift by linearly interpolating the YFP/CFP ratio in the buffer before and after stimulation. Although such interpolation might produce some error in determining the adaptation precision, inspection of the data showed that this error was significantly smaller than the variability between individual measurements.

### Simulation of Bacterial Behavior in Gradients

The effect of imprecise adaptation on bacterial navigation was studied with RapidCell program, v. 1.4.2 (http://rapidcell.vladimiroff.info). In each case, a homogeneous population of 4000 cells was simulated in a radially symmetric Gaussian gradient of attractant 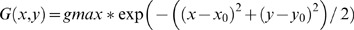 with the peak concentration *gmax* in the center of the field (*x_0_* =  *y_0_* = 3 mm). Cells were released from the left wall (*x* = 0.02 mm, *y = *3 mm) and the mean *x*-position of the population <*x*(*t*)> as well as the distribution of *x*-positions of individual cells around the mean were tracked for 2000 s. The relative adaptation rate was set to 0.4 to match the FRET experimental data for adaptation time.

For simulations with fixed precision of adaptation, the level of CheY-P was described as 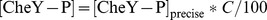, where [CheY-P]_precise_ is the normalized CheY-P concentration in precisely adapting cells [12], and *C* is the adaptation precision in %. Concentration-dependent precision of adaptation was obtained from experimental data shown in Fig. 1B and described as 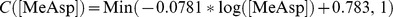, where [MeAsp] is the MeAsp concentration in mM.

## Supporting Information

Figure S1 **Mean position <** ***x*** **(** ***t*** **)> as function of time for cells with a concentration-dependent precision of adaptation.** Simulation was performed as in Fig. 2D but in gradients with a *gmax* of 0.1, 1 or 100 mM MeAsp. Solid lines indicate the mean <*x*(*t*)>, thin dotted lines indicate the distribution of the population around the mean, <*x*(*t*)> − s. d. and <*x*(*t*)>+s. d., respectively.
(TIF)

Figure S2 **Inhibition of** ***E. coli*** **growth by serine.** **(A)** Relative growth rate of MG1655 cells grown at 34°C in M9 minimal medium supplemented with 0.4% glycerol and four amino acids. 1 mM of each indicated amino acid was added to the culture after 2 hours of growth. The relative growth rate was determined as in Fig. 3. **(B)** Relative growth rates of indicated strains, measured as in (A) but in presence of varying concentrations of serine.
(TIF)
