# Supplementary figures and images for: Imprecision of Adaptation in Escherichia coli Chemotaxis
Source: PLoS One. 2014 Jan 8;9(1):e84904. doi: 10.1371/journal.pone.0084904 (PMC3885661; doi:10.1371/journal.pone.0084904)

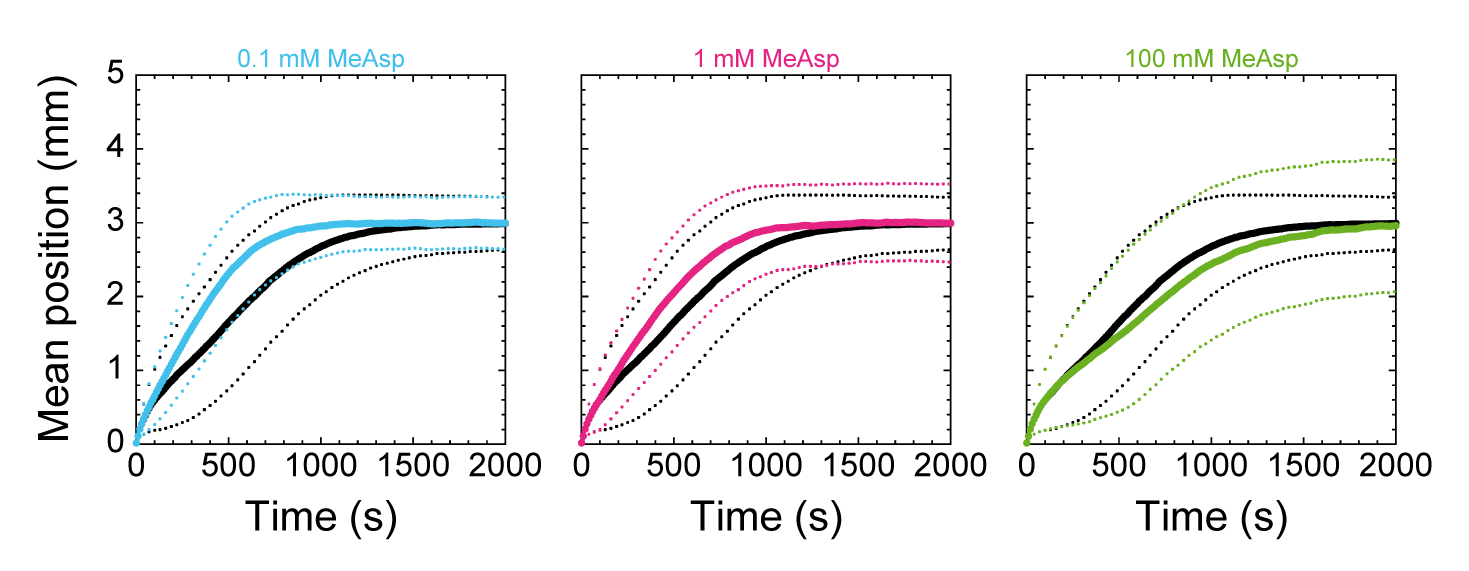

Supplement: Figure S1 — Mean position < x ( t )> as function of time for cells with a concentration-dependent precision of adaptation. Simulation was performed as in Fig. 2D but in gradients with a gmax of 0.1, 1 or 100 mM MeAsp. Solid lines indicate the mean <x(t)>, thin dotted lines indicate the distribution of the population around the mean, <x(t)> − s. d. and <x(t)>+s. d., respectively. (TIF) [file pone.0084904.s001.tif]

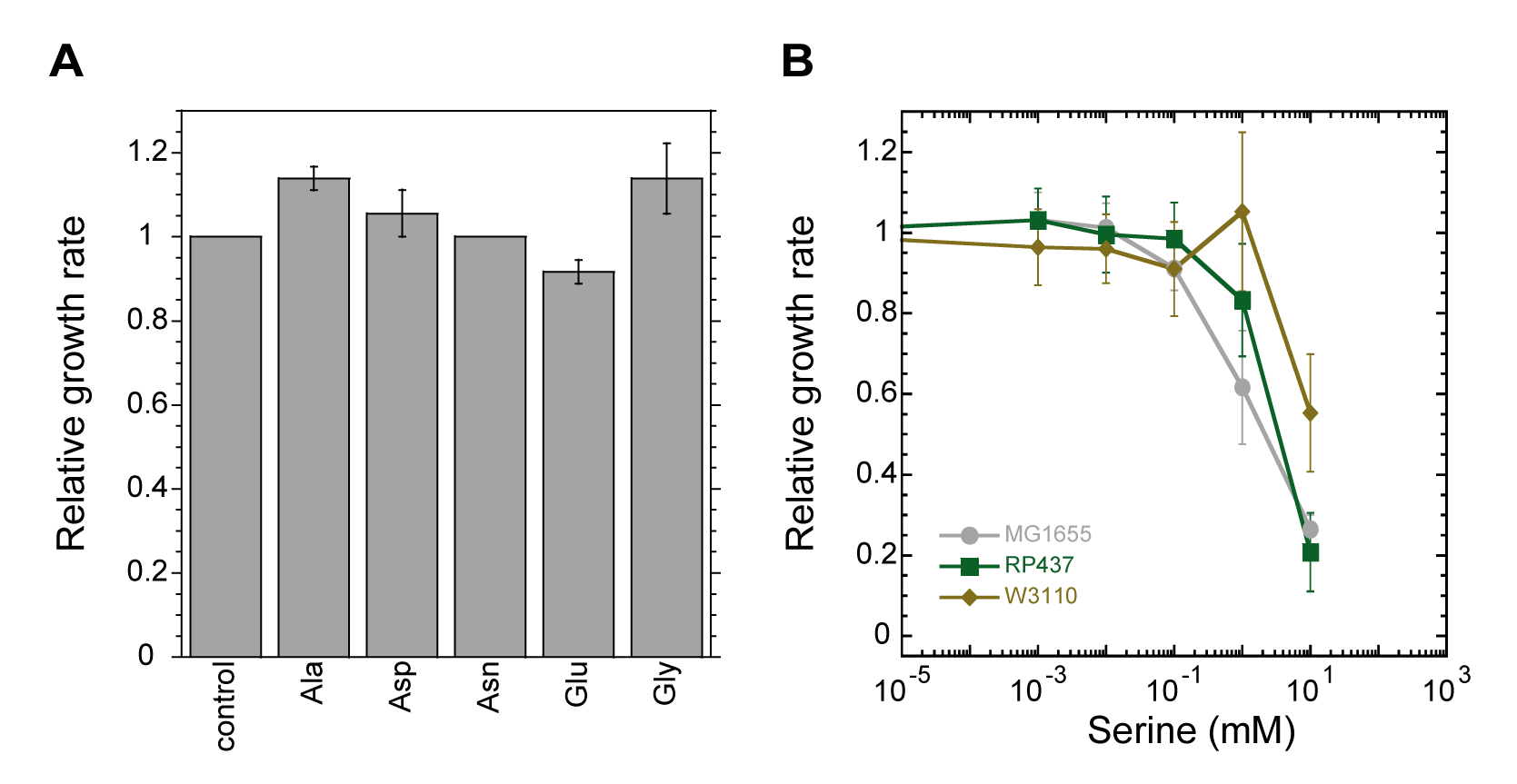

Supplement: Figure S2 — Inhibition of E. coli growth by serine. (A) Relative growth rate of MG1655 cells grown at 34°C in M9 minimal medium supplemented with 0.4% glycerol and four amino acids. 1 mM of each indicated amino acid was added to the culture after 2 hours of growth. The relative growth rate was determined as in Fig. 3. (B) Relative growth rates of indicated strains, measured as in (A) but in presence of varying concentrations of serine. (TIF) [file pone.0084904.s002.tif]
